# Origin of the electrocatalytic activity in carbon nanotube fiber counter-electrodes for solar-energy conversion†

**DOI:** 10.1039/d0na00492h

**Published:** 2020-08-10

**Authors:** Alba Martínez-Muíño, Moumita Rana, Juan J. Vilatela, Rubén D. Costa

**Affiliations:** IMDEA Materials Institute c/ Eric Kandel 2, Getafe 28906 Madrid Spain juanjose.vilatela@imdea.org; Universidad Autónoma de Madrid, Departamento de Física Aplicada Calle Francisco Tomás y Valiente, 7 28049 Madrid Spain; Technical University of Munich, Chair of Biogenic Functional Materials Schulgasse, 22 94315 Straubing Germany ruben.costa@tum.de

## Abstract

Carbon nanotubes are a versatile platform to develop sustainable and stable electrodes for energy-related applications. However, their electrocatalytic activity is still poorly understood. This work deciphers the origin of the catalytic activity of counter-electrodes (CEs)/current collectors made of self-standing carbon nanotube fibers (CNTfs) using Co^2+^/Co^3+^ redox couple electrolytes. This is based on comprehensive electrochemical and spectroscopic characterization of fresh and used electrodes applied to symmetric electrochemical cells using platinum-based CEs as a reference. As the most relevant findings, two straight relationships were established: (i) the limiting current and stability increase rapidly with the surface concentration of oxygen-containing functional groups, and (ii) the catalytic potential is inversely related to the amount of residual metallic Fe catalyst nanoparticles interspersed in the CNTf network. Finally, the fine tuning of the metal nanoparticle content and the degree of functionalization enabled fabrication of efficient and stable dye-sensitized solar cells with cobalt electrolytes and CNTf-CEs outperforming those with reference Pt-CEs.

## Abstract 2

A study of the role of functional groups and residual Fe catalyst on the high activity of carbon nanotube (CNT) fibre counter electrodes outperforming Pt in dye-sensitised solar cells (DSSCs) with Co^2+^/Co^3+^ redox couple electrolytes.

## Graphical abstract

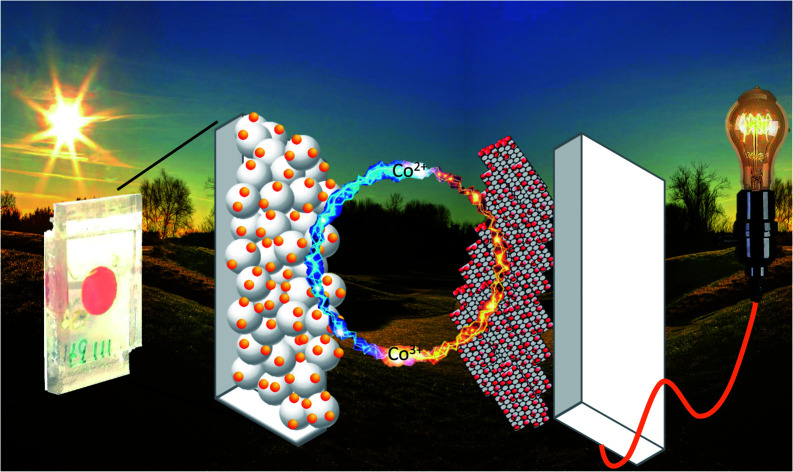

## Introduction

Carbon nanotubes (CNTs) are an attractive material for sustainable and highly efficient electrodes in electrocatalytic energy-related applications.^1–3^ However, there are still uncertainties regarding the origin of their catalytic activity with respect to surface functionalization, type of CNT/entanglement, and catalyst impurities. For instance, CNTs are generally synthesized using a metal catalyst, such as Fe, Cu, or Ni.^4–6^ The residual catalyst can reach >30% wt,^7^ most often as metal nanoparticles, but also as carbides and/or oxides.^7,8^ It is either encapsulated in the CNT network or as interspersed nanoparticles capped with a graphitic shell. To date, the presence of metallic impurities has scarcely been recognized as critical for electrocatalytic processes.^9^ In contrast, there has been much more research on the electrocatalytic behavior of CNTs in terms of type of CNT,^3,10^ functionalization degree and/or defects,^11^ and CNT alignment.^12,13^

Despite several efforts, the role of defects and impurities in CNT-based electrodes for solar energy conversion processes is still elusive.^1,2^ This is especially critical for dye-sensitized solar cells (DSSCs), in which the electrochemical regeneration of the dye and the electrolyte is the key process.^14,15^

To date, the DSSC field has reached a mature state, achieving energy conversion efficiencies (*η*) superior to 14% (outdoor)^14^ and 28.9% (indoor).^15^ There is still extensive research focused on: (i) increasing device efficiency and stability,^14,15^ (ii) replacing unsustainable materials, such as Pt in counter-electrodes (CEs) and iodine electrolytes,^16–18^ and (iii) enabling augmented mechanical properties.^19,20^ In this context, CNT electrodes are becoming key to tackling these challenges towards highly efficient DSSCs as they combine (i) ultrafast exciton/charge transfer (1–10 ps),^21–25^ (ii) extraordinarily high mobility (10^5^ cm^2^ V^−1^ s^−1^),^26^ (iii) high electrochemical stability,^27–29^ (iv) mechanical features when assembled as macroscopic CNT fibres or fabrics (CNTfs),^30,31^ and (v) simple, rapid, and inexpensive processing as large-area structures.

CNTs, and specially MWCNTs, reduce the charge-transfer resistance of DSSCs, along with providing a good electrode–electrolyte interface and a high specific surface area, which is mainly reflected in FF and efficiency values.^32,33^ Indeed, wire-like DSSCs with CNTf-CEs and a TiO_2_ photoanode have reached *η* > 10% as wearable devices.^31^ In contrast, planar architectures had commonly achieved low efficiencies with an I^−^/I_3_^−^ redox electrolyte,^34,35^ until we recently demonstrated that highly graphitic and crystalline CNTfs used as current collectors/CEs realize an *η* of *ca.* 9%.^36^

Improving the performances of CEs is still a challenge, and composites based on carbon materials (such as CNTs, carbon nanofibers – CNF, graphene, *etc.*) and transition metal compounds (TMCs) are actively researched candidate materials. These composites generally improve the electrochemical performance of TMCs and the electrocatalytic activity towards a triiodide reaction, such as in the case of CNF–Pt composites.^37–39^ On the other hand, they tend to present a lower charge-transfer resistance (compared to Pt) and high surface area,^40–44^ derived from the carbon material. Despite their promising performance as CEs,^34–36,45–48^ the origin of the catalytic behavior of CNTf-electrodes (or their composites) is also poorly understood in DSSCs. Current gaps in our understanding of the origin of the catalytic activity have, in addition, restricted their use to iodine-based electrolytes, when in fact carbon derivatives are known to be highly efficient catalysts for many other redox couples and hole transport materials (HTMs).^49–51^

In this context, this work sets out to decipher the origin of the catalytic behavior of CNTfs in terms of surface functionalization and metallic impurities using emerging electrolytes (Co^2+^/Co^3+^ redox)^52^ for highly efficient planar DSSCs. This rationale is supported by a comprehensive spectroscopic and electrochemical study of fresh and used CNTf-electrodes applied to both symmetric cells and fully operational DSSCs. As the most relevant findings, two straight relationships are established: (i) the limiting current and stability increase dramatically with the degree of oxidative CNTf functionalization, and (ii) the catalytic potential is inversely related to the amount of the residual metallic Fe catalyst interspersed in the CNTf network. Hence, metallic impurities are the catalytic centers, while surface functionalization determines the limiting current. Based on these findings, we further optimized the CNTf-electrodes to achieve highly efficient and stable DSSCs, outperforming those with reference Pt-CEs.

Overall, this work clearly demonstrates that CNTf-CEs featuring metallic Fe impurities lead to highly performing multifunctional electrodes – *i.e.*, electrocatalytic electrolyte regeneration, and acting as a current collector – beyond using traditional redox couples in DSSCs. This paves the way towards future work focused on copper(i) complex electrolytes,^15,53^ thiolates/disulphides^54–56^ for tandem solar cells, and polysulfides for quantum dot sensitized solar cells.^57,58^

## Experimental

### Materials

Thiophene (extra purity ≥99%) and ferrocene (purity = 98%) were obtained from Acros Organics and 2-butanol (purity > 99%) from Sigma Aldrich. Ferrocene was purified by a sublimation/recrystallization process. The D35 organic dye (DN-F04) and the [Co(bpy)_3_][TFSI]_2_/[Co(bpy)_3_][TFSI]_3_ redox couple used for the preparation of a cobalt-based electrolyte were purchased from Dyenamo AB (Sweden). 1-Butyl-3-methylimidazolium iodide, guanidine thiocyanate, 4-*tert*-butylpyridine, iodine, acetonitrile (ACN) and valeronitrile were purchased from Sigma Aldrich. Transparent conductive glass substrates were obtained from XOP Glass (FTO, 15 Ω per square). TiO_2_ 18NR-AO Active Opaque Titania Paste and 18NR-T Transparent Titania Paste were purchased from Dyesol UK. TiCl_4_ was bought from Fisher Scientific (titanium(iv) chloride solution, 0.09 M in 20% HCl). CNTfs with effectively 0% metal content were purchased from DexMat.

### CNTf synthesis

The CNTfs were synthesized by the direct spinning floating catalyst chemical vapor deposition (CVD) method^59^ using butanol as the carbon source, ferrocene as the Fe catalyst source and thiophene as the promoter, at a concentration of 0.8 wt% ferrocene, 1.5 wt% thiophene and 97.7 wt% butanol adjusted so as to produce long, highly graphitic CNTs of a few layers.^60^ The reaction was carried out in a hydrogen atmosphere at 1250 °C in a vertical furnace, using precursor feed rates 5 mL h^−1^ and a fiber winding rate of 7–9 m min^−1^. The electrodes were produced by directly winding multiple CNT filaments on a paper substrate so as to form a unidirectional non-woven fabric. The fabric is a highly aggregated porous material with high mechanical robustness. This material was transferred to FTO-glass using a simple press-transfer technique to fabricate electrodes with a thickness of around 10 μm for DSSCs. Purification of the CNT fabric was performed by heating the samples at high temperature followed by acid treatment.^61^ Fibres of DWCNTs with ultra-high purity were obtained from the commercial supplier Dexmat. These fibers were produced by wet-spinning from a liquid crystalline dispersion of high-crystallinity predominantly double-walled carbon nanotubes DWCNTs.

### Device fabrication

After following the standard washing procedure,^62^ each electrode was subjected to UV–O_3_ treatment for 18 min (Model No. 256e220, Jelight Company, Inc). The pre-cleaned FTO substrates were pre-treated by immersion in a 45 mM TiCl_4_ solution (titanium(iv) chloride solution, 0.09 M in 20% HCl from Sigma), in order to suppress the electron recombination, and annealed at 400 °C (30 min). After cooling down, an opaque film of TiO_2_ paste (18NR-AO Active Opaque Titania Paste) or 2 layers of transparent TiO_2_ paste (18NR-T Transparent Titania Paste) were doctor bladed on the electrodes. Right after, the electrodes were annealed at 325 °C (5 min), 375 °C (5 min), 450 °C (15 min), and at 500 °C (15 min). The resulting films were post-treated with 20 mM TiCl_4_ aqueous solution for 60 min at 70 °C and re-annealed at 450 °C (60 min), resulting in a thin film of around 10 μm, as measured by using a stylus profilometer (KLA-Tencor, Alpha-Step D500). The CNTf-CEs were prepared by press transferring CNTf strips on pre-cleaned FTO glass, condensing with drops of pure ethanol and drying at 70 °C for 15 min, reaching a thickness of 10 μm. For the Pt electrodes used as a reference, a 5 mM solution of hexachloroplatinic acid in isopropanol was drop cast on pre-cleaned FTO substrates and annealed at 400 °C for 20 min. The sealing of the devices was performed using a spacer of 50 μm thickness. The photoanodes were further sensitized overnight by immersion in a 0.2 mM [(*E*)-3-(5-(4-(bis(2′,4′-dibutoxy-[1,1′-biphenyl]-4-yl)amino)phenyl)thiophen-2-yl)-2-cyanoacrylic acid (D35) – Dyenamo] ethanol solution. A cobalt-based electrolyte consisting of 0.25 M [Co(bpy)_3_][TFSI]_2_, 0.06 M [Co(bpy)_3_][TFSI]_3_, 0.1 M bis-trifluoromethane-sulfonimide lithium salt (LiTFSI), and 0.5 M 4-*tert*-butylpyridine (4-TBP) in acetonitrile (ACN), was injected between the electrodes. The symmetrical cell configuration that was used for the electrochemical characterization consisted of two identical electrodes of CNTfs or Pt, separated using a spacer (100 μm) and incorporating the cobalt electrolyte. The device figures-of-merit discussed in this work were an average of up to 3 devices, having a standard deviation of *ca.* 0.7%.

### Instrumentation

Raman analysis was performed on a Renishaw inVia Reflex spectrometer with an Ar^+^ ion laser (*λ* = 532 nm) excitation source. The laser light was focused on the samples using a 100 objective lens at a power of around 1.6 mW in order to avoid sample heating. Spectral deconvolution was carried out by non-linear least square fitting of the Raman peaks to Lorentzian line shapes. Cyclic voltammograms (CVs) were recorded on thin layer symmetrical cells made of identical electrodes, filled with the electrolyte, in an electrode/electrolyte/electrode configuration. CV experiments were conducted using an Autolab PGSTAT-30 potentiostat (Ecochemie) with a scan rate of 20 mV s^−1^ and a voltage range from −1 to +1 V *versus* the electrode. The stability studies were carried out before and after repetitive CV cycles (200) monitoring the changes in the Raman spectra of the CNTfs.

The diffusion coefficient (*D*_Co^3+^_) of the reaction determining species (Co^3+^), in the electrolyte bulk has been calculated from the *J*–*V* curves of the CV experiments in the region of the limiting current, using the following equation of Fick's diffusion law:*D*_Co^3+^_ = *J*_lim_*δ*/*nFC*where *n* = 1 is the number of exchanged electrons during the redox reaction, *C* is the initial concentration of the determining species, *δ* is the distance of the electrodes, and *F* is the Faraday constant.

The theoretical diffusion constant was calculated using the Stokes–Einstein relation:*D*_Co^3+^_ = *k*_b_*T*/6π*ρ*_ACN_*r*_Co^3+^_with *k*_b_ being the Boltzman constant, *T* the temperature, *ρ*_ACN_ the viscosity of ACN at 298 K and *r*_Co^3+^_ the radius of Co(bpy)_3_^3+^.^63^ The theoretical current density was calculated with the Randles–Sevcik equation:*J* = 0.4463*nFC*(*nFvD*/*RT*)^1/2^where *n* is the number of exchanged electrons, *F* is the Faraday constant, *C* is the concentration, *v* is the scan rate velocity, *D* is the diffusion constant and *R* is the universal gas constant. Current density–voltage (*J*–*V*) measurements were performed by illuminating the DSSCs (cell structure: FTO/compact layer/mesoporous TiO_2_/D35/Co^3+^/Co^2+^ electrolyte/CNTf/FTO) using solar simulated light (1 sun, 1000 W m^−2^) calibrated with a KG5-filtered Silicon reference cell (Newport/Oriel, Model 91150V) from a 150W-Xe source (Oriel), in combination with an AM 1.5G optical filter. The active area of the DSSCs was set at 0.09 cm^2^, using a large black mask in front of the cells in order to avoid any light piping inside the cell. The *J*–*V* characteristics were recorded using linear sweep voltammetry with the Autolab potentiostat working in a 2-electrode mode at a scan rate of 50 mV s^−1^. Photoelectrochemical and photovoltaic characterization was performed on a batch of at least three cells for each electrode, and the mean value (without a significant deviation) for the obtained results was derived. The results from electrochemical and photoelectrochemical measurements were deduced from the cells that presented performance closest to the average. Electrochemical impedance spectra (EIS) were recorded on the cells using the same potentiostat, equipped with a frequency response analyzer (FRA), at 0 V *vs.* the electrode under dark and light conditions recorded over a frequency range of 100 kHz to 10 mHz. The obtained spectra were fitted with FRA software provided by Autolab in terms of appropriate equivalent circuits.

## Results and discussion

### The role of functional groups

**Fig. 1:**
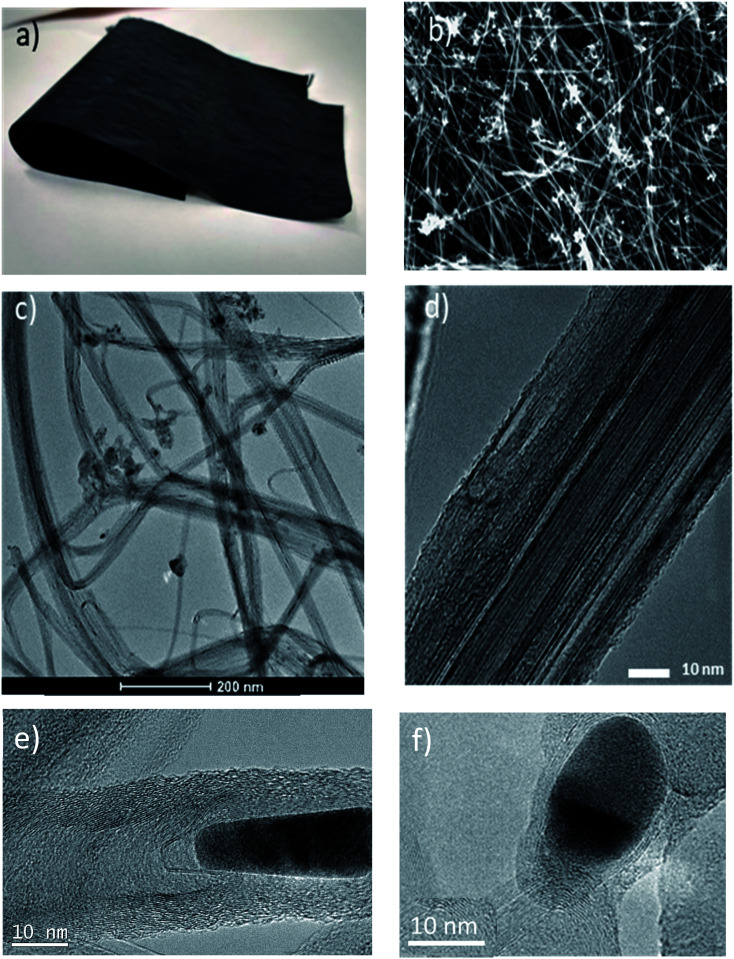
Digital photograph (a), and SEM (b), and low magnification TEM (c) images showing the large porosity of a CNT and its impurities. The high magnification TEM (d) image highlights the high degree of graphitization of the few-layer CNTs. High resolution TEM (HRTEM) images of encapsulated Fe impurities (e and f).

The CE material is macroscopic fabrics of CNTs produced by directly spinning a continuous fibre from the gas phase during CNT synthesis – see the Experimental section for more details. Fig. 1 shows that the internal structure of the fabrics consists of an open network of CNT bundles giving rise to mesopores (10–50 nm) and a high specific surface area (with values above 250 m^2^ g^−1^).^64^ In addition to CNTs, the samples have non-graphitic carbon impurities and 9 wt% residual catalyst nanoparticles distributed throughout the fabric and encapsulated along the fibre, as shown in Fig. 1e and f. These nanoparticles consist of an Fe core (predominantly metallic γ-Fe) and a continuous encapsulating shell of graphene.^65^ They show no preferential faceting. The CNTs are exceptionally long (1 mm), and have a few layers, and a relatively high degree of perfection – see Fig. 1d and Raman spectra in Fig. S1.†

To gain insight into role of surface defects, a set of samples were functionalized through ozonolysis for different times time.^66,67^ The method preserves the highly conducting network, while enabling controlled introduction of oxygen containing groups, such as C–O, O–C=O, COOH and C–OH on the graphitic surface of the material, leading to a higher O concentration compared to that of the pristine material.^60,66^ Other superficial defects, such as holes and broken layers, appear as a consequence of the transformation of C=C bonds to out of plane C–C bonds in the new functional groups. Ozonolysis also removes surface impurities with no detectable effect on the residual catalyst nanoparticles.^66^

The degree of functionalization can be readily monitored by Raman spectroscopy: a drop in absolute intensity due to resonance loss, the appearance of the D′ mode (double resonance induced by defects and disorder) and the increase of the intensity ratio of the D and G bands (*I*_D_/*I*_G_)^68^ are the main characteristics. Here, *I*_D_/*I*_G_ increases from 0.64 ± 0.08 for the pristine material (1), to 0.80 ± 0.05 (2), and to 1.38 ± 0.05 (3) for the samples functionalised for 2 and 4 minutes, respectively. The XPS spectrum of functionalized fibres shows an increase in the O 1s region, along with an increase in the O1s/C1s ratio, due to the formation of oxygen functional groups. The corresponding changes in surface energy and elemental O/C concentration can be found in references.^56,68^

**Fig. 2:**
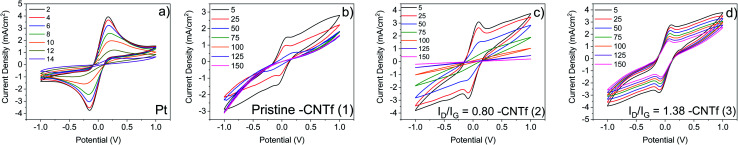
LSV experiments of symmetric cells with Pt (a) and CNTf electrodes: 1 (b), 2 (c) and 3 (d).

These CNTf samples were transferred onto glass substrates and were applied as electrodes for symmetrical cells with a CNTf/cobalt-based electrolyte/CNTf configuration – see the Experimental section for details. The electrocatalytic activity of pristine and functionalized CNTf-CEs was studied using linear sweep voltammetry (LSV). Symmetrical cells with Pt-electrodes are provided for reference purposes. In all the cases, the reference electrode was short circuited with the working electrode. Similar to what has been described using iodine-electrolytes,^32–34^ LSV curves are almost symmetrical with respect to oxidation/reduction peaks. Fig. 2 shows that 1-cells present slightly better electrocatalytic behavior towards the Co^2+^ → Co^3+^ redox reaction than that of Pt-cells as lower redox potentials and similar limiting current densities (*J*_lim_) are noted – Table 1.

**Table 1:** Spectroscopic and electrochemical characterization of the electrodes

Electrodes	*I*_D_/*I*_G_^a^	Impurity content (%)	*V*_red_ (V)	*V*_ox_ (V)	*J*_lim_ (mA cm^−2^)	*D*_Co^3+^_ × 10^−6^ (cm^2^ s^−1^)
1	0.64	≈9	−0.057	0.089	1.536	2.65
2	0.80	≈9	−0.079	0.101	2.577	4.45
3	1.38	≈9	−0.097	0.103	2.615	4.52
Pt	—	—	−0.145	0.179	1.517	2.62

a Ratio between the intensity of the D peak and the G peak.

This further enhances upon functionalizing the CNTfs. For instance, 2- and 3-cells feature redox potential values that hold almost constant, while the *J*_lim_ and *D*_diff_ values are almost 2-fold higher than those for 1 and Pt-cells – Table 1. Interestingly, 2- and 3-cells exhibit similar voltammograms, suggesting that the enhanced performance is related to an increase of the surface area due to the formation of new defects and pores.^69^ Indeed, the *D*_diff_ values of *ca.* 4.5 × 10^−6^ cm^2^ s^−1^ are very close to the values obtained in the literature for highly efficient devices based on liquid electrolytes.^43,60–65^ This suggests that the electrodes are well-optimized; the typical mass transport limitation caused by the bulky nature of the Co^2+^/Co^3+^ complexes is circumvented with the functionalisation of the CE. Regarding the electrochemical impedance spectroscopy study (EIS), 1-cells (Fig. S3†) present two semicircles of ≈250 Ω and >400 Ω in the Nyquist plot centered at 10^3^ to 10^4^ Hz, corresponding to the charge transfer resistance (*R*_ct_) and at 10^−2^ to 1 Hz, corresponding to the bulk diffusion of the electrolyte (*R*_diff_), respectively. These contributions are highly increased after the LSV experiments, confirming the disappearance of the activity. As the functionalization time increases, *R*_ct_ and *R*_diff_ remain centered at 10^4^ Hz and 10^−2^ Hz, with less variation after the LSV experiments (Fig. S4†), supporting the stability increase seen in the LSV.

**Fig. 3:**
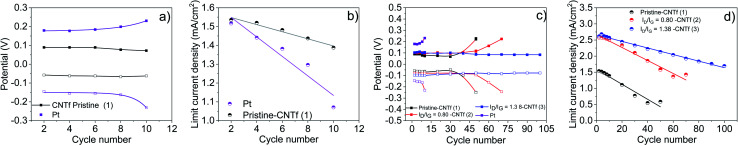
Reduction (empty squares) and oxidation (filled squares) potentials (a and c) and limiting current densities (b and d) *versus* the number of cycles of symmetric cells with Pt (purple), 1 (black), 2 (red) and 3 (blue). Lines in (b) and (d) correspond to the fittings of the limiting current densities.

In general, CEs must combine high electrocatalytic activity and electrochemical stability, though this has been difficult when catalytic activity originates from defects in carbon-based CEs.^16–18,27,28^ In addition, the stability issue is even more critical in cobalt-based electrolytes, as they are prone to degrade.^42,43,60–65^ In this line, the low stability of Pt-cells is manifested by an increase of the redox potential and a decrease of *J*_lim_ after 10 LSV cycles – Fig. 2 and 3. This is typically ascribed to the interaction with *tert*-butyl pyridine (TBP) and the ligand dissociation in ACN^74–77^ along with Pt poisoning by air exposure.^42,43,60–65^

In contrast, 1-cells feature stable redox potentials over 30 cycles, showing a loss of *J*_lim_ of <60% with respect to that of the fresh electrodes – Fig. 2. Surprisingly, the stability is further enhanced upon functionalization. For instance, 3-cells retain stable redox potentials after 100 LSV cycles, while maintaining a high *J*_lim_.

Fig. 3 gathers the changes in the redox potential and *J*_lim_ as a function of the number of cycles (*N*_c_), highlighting (i) the higher electrochemical stability of CNTf CEs compared to Pt, and (ii) the large increase in *J*_lim_ and its retention upon functionalization. Fitting of the experimental data provides simple metrics to compare the different electrodes – Table 2.

**Table 2:** Empirical dependence of the redox potential and limiting current density on the cycle number

Electrode	Oxidation potential (*V*_ox_ = *V*_ox0_ − *a*e^*bN*_c_^)	Limiting current density (*J*_lim_ = *J*_lim0_ − *c* × *N*_c_)
1	−0.060 − 2 × 10^−5^e^0.18 × *N*_c_^; *R*^2^ = 0.98	1.59 − 0.023 × *N*_c_; *R*^2^ = 0.95
2	−0.079 − 1 × 10^−4^e^0.10 × *N*_c_^; *R*^2^ = 0.97	2.66 − 0.0201 × *N*_c_; *R*^2^ = 0.97
3	−0.096 − 2.2 × 10^−4 ×^*^N^*_^c^_; *R*^2^ = 0.80	2.66 − 0.0102 × *N*_c_; *R*^2^ = 0.99

The redox potential remains fairly linear and dramatically changes at the onset of the cell degradation. Functionalization reduces the associated exponent, reaching purely planar behavior in the highest functionalization sample – *i.e.*, 3-cells. *J*_lim_ is found to decrease linearly with the cycle number, but with an increasing slope with reduced functionalization of 0.0102, 0.201 and 0.23 mA per cm^2^ per cycle for 3, 2, and 1-cells, respectively.

The enhanced electrochemical stability and the high current density of CNTf-CEs upon functionalization could be related to two possible mechanisms: (i) electrochemical degradation of the electrode promoting the formation of more catalytic centers – *i.e.*, surface defects, and (ii) the increased number of charge transfer events and weaker interaction of the bipyridine ligand with the functionalized CNTf surface. First, the stability of the CNTf-CEs themselves was studied by Raman spectroscopy – see the Experimental section for details. Fig. S2† shows no significant changes in the peak position and the intensity of the Raman spectral features of CNTf-electrodes remains the same before and after electrochemical stress, regardless of the degree of functionalization. This evidences the lack of electrode degradation after operation in repetitive cycles, highlighting their exceptional stability. Indeed, the latter is also confirmed, since the redox potential did not change upon functionalization, while 2- and 3-cells featured, in addition, similar *J*_lim_ values – Table 1. The second and most likely reason for the increased current density and the electrocatalytic stability is the presence of defects in the CNTfs. Along with the high surface area, the presence of oxygenated functional groups in the ozonized CNTfs might contribute to the decrease in the activation energy of the electrocatalytic redox process of [Co(bipy)_3_]^3+^/[Co(bipy)_3_]^2+^, thereby increasing the current density. Such instances are widely established theoretically as well as experimentally for the iodide/triiodide redox pair.^78,79^

On the other hand, the redox reaction of the [Co(bipy)_3_]^3+^/[Co(bipy)_3_]^2+^ pair is an outer sphere electron transfer process, where the strength of the metal–ligand bond is strongly affected during the electron transfer (Co(ii): (t^2^_g_)^5^(e_g_)^2^ to Co(iii): (t^2^_g_)^6^(e_g_)^0^).

Since this catalytic electron transfer process occurs in the proximity of the graphitic layers of the CNTs, it is highly probable that the bipyridine ligands can interact with the CNT surface by π–π interaction and stabilize irreversibly.^80,81^ The origin of this process can be compared with the fact of selective separation of small molecules with available π-clouds using CNTs by taking advantage of the π electron rich, high surface area of the CNTs.^82,83^ Now the possibility of such non-covalent interaction of the bipyridine ligand with the CNT surface decreases when the outermost walls of the CNTs contain some defects (*e.g.* oxygen functional groups) making the graphitic surface very uneven for irreversible stabilization of the bipyridine ligands. This in turn possibly makes the redox process of [Co(bipy)_3_]^3+^/[Co(bipy)_3_]^2+^ more reversible on the ozonized CNTfs compared to the pristine CNTfs. Note that an increase in surface functional groups – *i.e.* in *I*_D_/*I*_G_ – produced an exponential increase in *J*_lim_ (Fig. S5†) and a large increase in stability, but without substantial changes in electrochemical redox potential.

### Metal impurity effect

We investigated the impact of the metal impurities on the electrocatalytic behavior. The amount of impurities was reduced from 9% wt to 3% wt through acid treatment, followed by electrochemical etching – Fig. S6.†^61^ This procedure was adjusted to also produce a similar functionalization degree to that of the above best CNTf electrode (3) – Fig. S7.† This sample is labelled 3–3%. Further comparison was also made with a commercial CNTf sample with effectively 0% wt. metal impurities, which was also oxidized to reach the same functionalization degree – Fig. S8.† This was labelled 3–0%. Through LSV and EIS tests on symmetric cells with these samples, we could, therefore, study the effect of the residual metal catalyst content in the range of 9, 3, and 0% wt at nearly the same degree of functionalization.

**Fig. 4:**
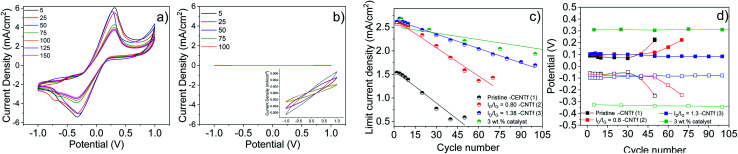
LSV curves of symmetric cells with 3–3% (a) and 3–0% (b) with an inset to clearly show the lack of redox features. Limiting current densities (c) and reduction (empty squares) and oxidation (filled square) potentials (d) *versus* the cycle number of symmetric cells with 1 (black), 2 (red), 3 (blue), and 3–3% (green) are shown. Lines in (c) are fittings of the limiting current densities.

As shown in Fig. 4, the decrease of the amount of impurities significantly impacts the electrocatalytic behavior of the CNTf-electrodes. In short, 3–3%-cells showed a 2-fold higher redox potential compared to 3-cells, indicating that the metal impurities present along the fibre play a key role as a catalytic center of the redox reaction. Importantly, the redox potential is stable over 100 LSV cycles while both, initial and loss *J*_lim_ values, are similar to those of 3. This points out that the functionalization is key towards enhanced electrocatalytic stabilities and current densities, while the Fe impurities are responsible for the electrocatalytic activity. This is further established using the featureless LSV curves of 3–0%-cells – Fig. 4, in which the Fe impurities are not present in the CNTfs. Importantly, these CNTf electrodes did not show any electrocatalytic activity in both pristine and functionalized forms – Fig. 4 and S9.† Regarding EIS tests (Fig. S10†), 3–3% cells show similar resistance values to those obtained with the functionalized samples 2 and 3, while the functionalized 3–0% symmetric cells show no-activity. This confirms that the type and amount of metallic impurities are key towards the future optimization of CNTf electrodes for energy-related applications.

### Solar cell characterization and performance

**Fig. 5:**
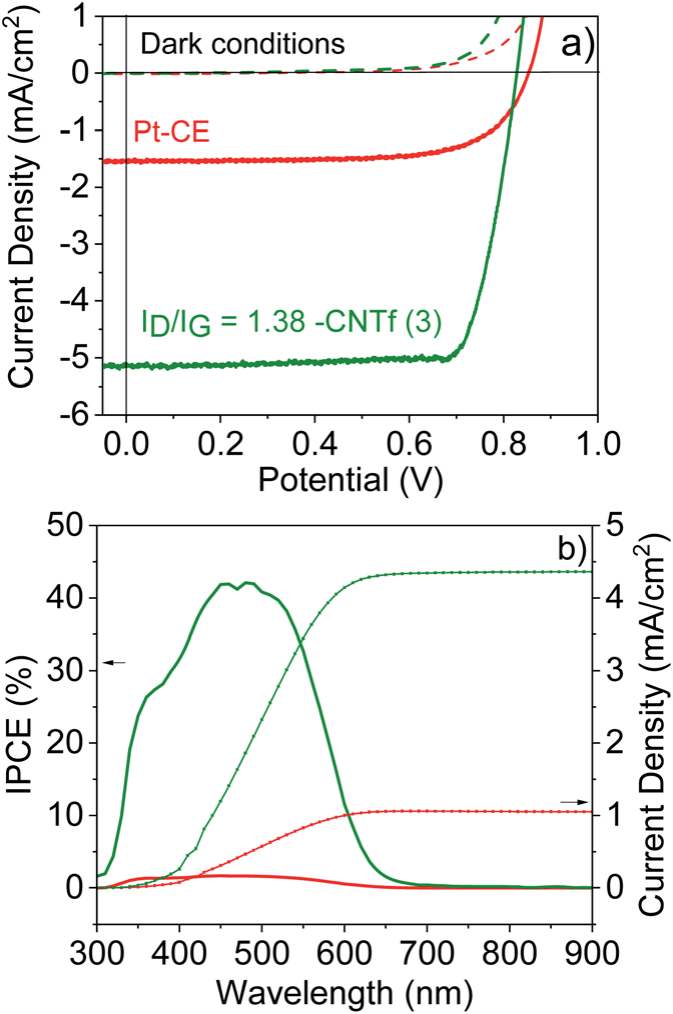
*J*–*V* characteristics (top) of the 3- (green) and Pt- (red) DSSCs under 1 sun illumination (solid line) and dark conditions (dashed lines) as well as IPCE and integrated current density (bottom).

Having determined the best configuration for CNTf-electrodes, we compare the performance of DSSCs with two different CEs: Pt as the reference and the optimum CNTf sample (3) – *i.e.*, highly functionalized and 9% wt metallic impurities. The DSSCs were fabricated with TiO_2_ mesoporous electrodes sensitized with an organic dye (D35) as photoanodes and a liquid cobalt-based electrolyte – see the Experimental section for details.^70–73^Fig. 5 displays the *J*–*V* curves and incident photon-to-current conversion efficiencies (IPCE) obtained under 1 sun AM 1.5G and dark conditions.

The devices using Pt-CEs exhibit a short circuit current (*J*_sc_) value of 1.55 mA cm^−2^ and a *V*_oc_ value of 0.85 V. The *V*_oc_ value is close to the theoretical one predicted for this cobalt-based electrolyte,^42^ while the IPCE matches the absorption of the dye, indicating that photogenerated currents are related to the photoexcitation of the dye. This goes along with fill factor (FF) values above 0.7, highlighting the quality of the solar cells.

The devices with 3-CEs feature similar device quality (FF = 70), but they exhibit a 5-fold increase in *J*_sc_ values. This is clearly in agreement with the results for symmetric cells. In line with devices using iodine-based electrolytes,^32–34^ the *V*_oc_ is, however, reduced. This could be related to the changes in the redox potential of the cobalt-based electrolytes due to different concentration gradients between the bulk electrolyte and the electrode surface as well as changes in the pH.^84^ Overall, the device efficiency increases from 1.5% (Pt-DSSCs) to 2% (3-DSSCs), highlighting the superior performance of the CNTf-CE for cobalt-based electrolytes.

EIS measurements were also carried out to gain further insight into the different processes taking place in the solar cells – Fig. S11.† The Nyquist plots consisted of only one or two semicircles centered at around 1–100 Hz and 1000–10 000 Hz. The former is related to the charge transport resistance of the photoanode, while the latter is related to the Warburg ion resistance. Therefore, we were unable to determine the resistance associated with the charge transfer (*R*_ct_) process at the electrolyte/CNTf-CE interface. This problem has already been encountered in devices based on iodine-based electrolytes.^36^

**Fig. 6:**
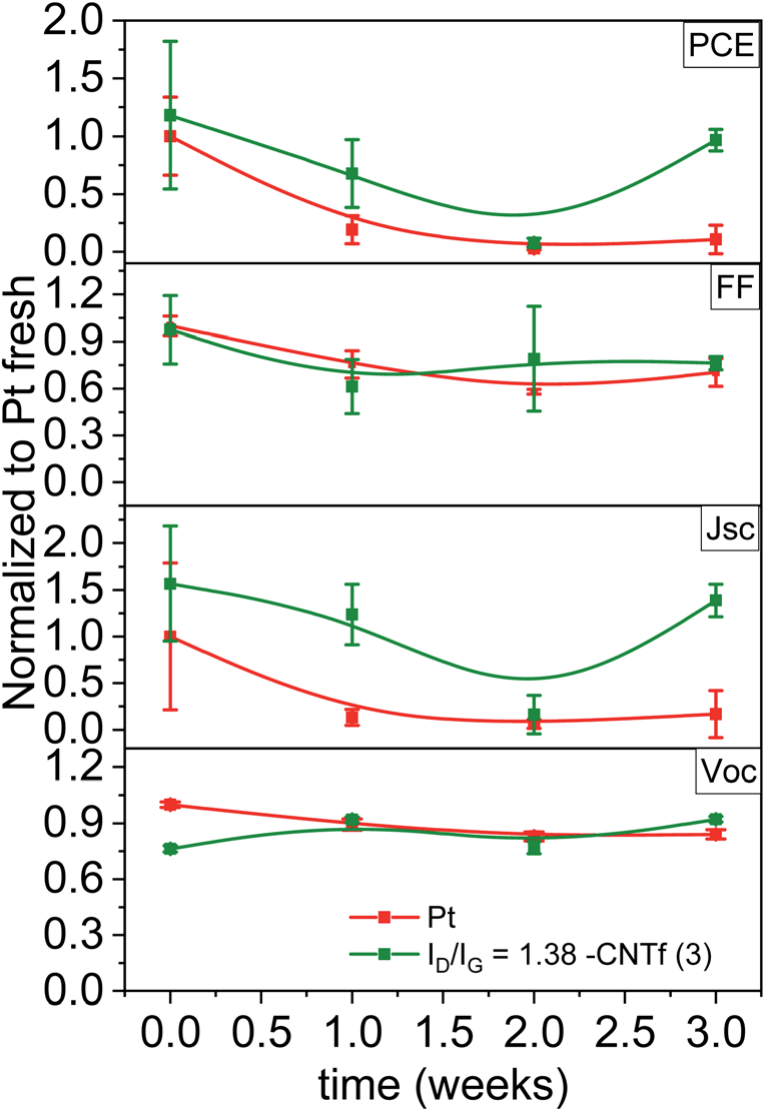
Changes of the figures-of-merit-of 3- (green) and Pt-(red) DSSCs over time during operation.

In line with the LSV results, Pt-DSSCs showed a loss in *J*_sc_ of >90% after 1 week, while *V*_oc_ and FF remained constant after 3 weeks – Fig. 6. In stark contrast, 3-DSSCs exhibited a notably superior stability. After 3 weeks under working conditions, both, *V*_oc_ and FF, remained constant, while *J*_sc_ slightly reduced by <10%.

## Conclusions

We have deciphered the origin of the electrocatalytic behavior of CNTf electrodes with the aim to use them as an alternative to Pt-CEs in DSSCs with a Co^2+^/Co^3+^ redox couple electrolyte. Similar to what has been observed in iodide-based electrolytes, the CNTf-CEs feature a significant catalytic activity towards Co^2+^/Co^3+^ redox couple electrolytes and can be optimized in terms of their functionalization and metal impurity content. On the one hand, upon CNTf functionalization through ozonolysis (*I*_D_/*I*_G_ = 1.38), higher limiting current densities and stabilities are realized in symmetric cells. On the other hand, metallic Fe impurities are key towards the catalytic behavior that is inexistent for CNTfs without metal catalysts and superior for CNTf electrodes with a higher amount of 9% wt. As such, we conclude that the CNTf surface chemistry has a significant effect on the adsorption process of the redox active species near the residual Fe impurities that act as catalytic centers. The high charge density and the number of the Fe-based centers are key for the charge transfer process, while the functionalization enhances the dynamics of the electrolyte regeneration. These findings were used to optimize the CNTf-electrodes for DSSCs. Here, the devices prepared with the optimized CNTf-CEs clearly outperformed those with Pt-CEs, showing, in addition, operational stabilities for several weeks. Further optimization of these devices requires an in-depth study of the storage (surface crystallization) and electrochemical stability of the Co-complex electrolytes using CNTf electrodes with respect to the type of counter-anion and ligand design.

Building on recent studies focused on devices with the standard I^−^/I_3_^−^ redox couple,^31–38^ we provide new insights into the most important aspects towards optimizing CNTf-electrodes for solar energy conversion devices using other redox electrolytes. Thus, we strongly believe that this work paves the way for using other electrolytes in CNTf-based DSSCs, in general, and other solar-driven energy applications using CNTf-electrodes, in particular.

## Conflicts of interest

There are no conflicts to declare.

## Supplementary Material

NA-002-D0NA00492H-s001
