# Supplementary material for: Origin of the electrocatalytic activity in carbon nanotube fiber counter-electrodes for solar-energy conversion
Source: Nanoscale Adv. 2020 Aug 10;2(10):4400–9. doi: 10.1039/d0na00492h (PMC9417869; doi:10.1039/d0na00492h)
Supplement: NA-002-D0NA00492H-s001 [file NA-002-D0NA00492H-s001.pdf]

Electronic Supplementary Material (ESI) for Nanoscale Advances.
This journal is © The Royal Society of Chemistry 2020

Supplementary information

Origin of the electrocatalytic activity in carbon nanotube fiber counter-electrodes for solar-energy conversion

Alba Martínez-Muíño[ab], Moumita Rana[a], Juan J. Vilatela [a] and Rubén D. Costa[ac]**

[a]IMDEA Materials Institute, c/ Eric Kandel 2, Getafe, 28906, Madrid, Spain E-mail: juanjose.vilatela@imdea.org

[b]Universidad Autónoma de Madrid, Departamento de Física Aplicada, Calle Francisco Tomás y Valiente, 7, 28049, Madrid, Spain

[c]Technical University of Munich, Chair of Biogenic Functional Materials Schulgasse, 22, 94315, Straubing, Germany. E-mail: ruben.costa@tum.de

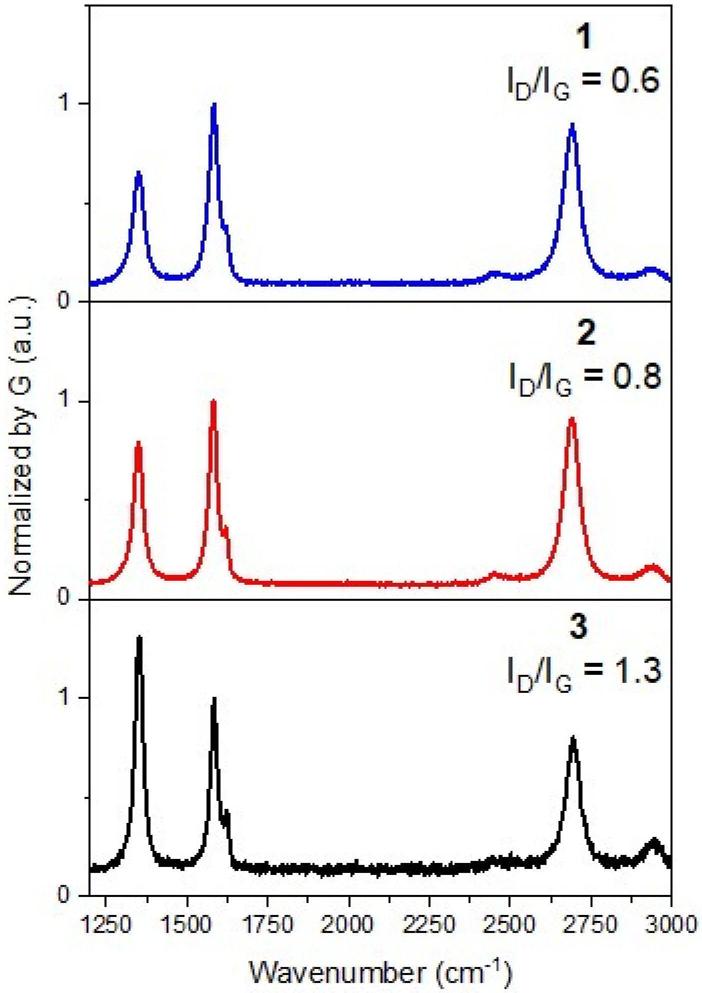

Figure S1. Raman characterization of CNTf electrodes upon ozonolysis treatment.

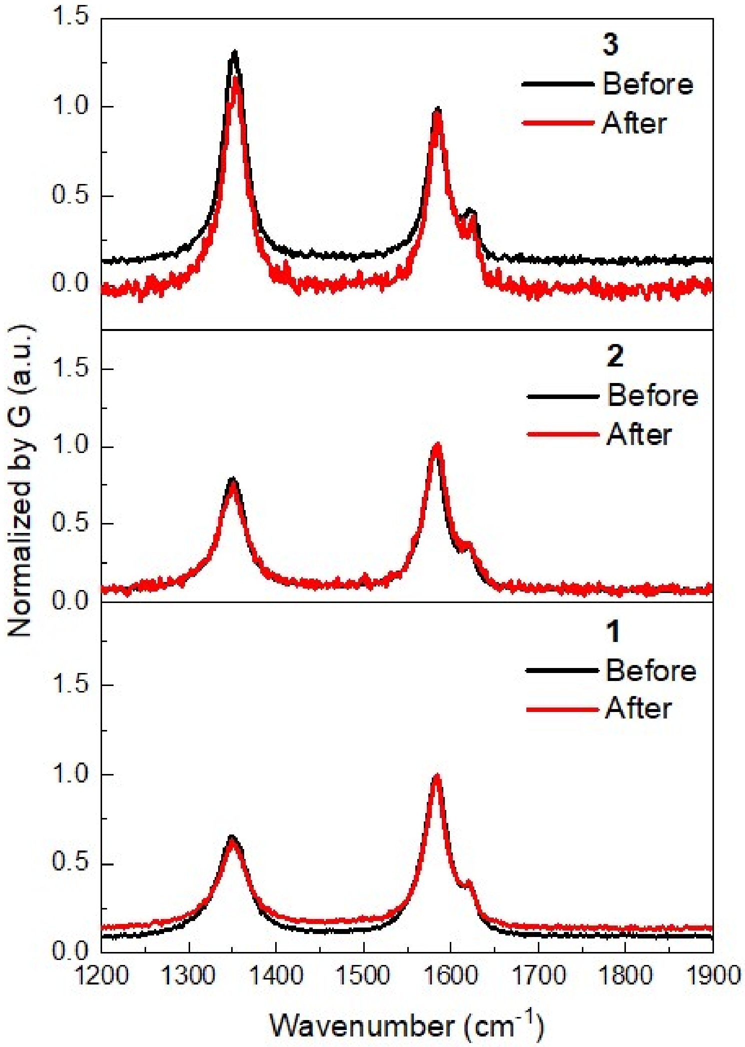

Figure S2. Raman spectra of the CNTf-electrodes before and after subjecting them to LSV assays in symmetric cells.

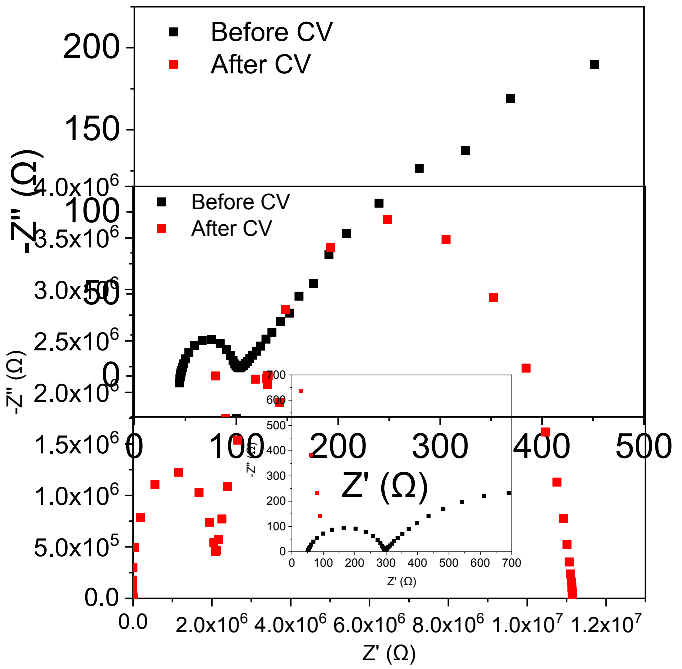

Figure S3. Nyquist plots of Pt (top) and **1** (bottom) symmetric cells before (black) and after (red) CV. Inset: zoom of the Nyquist plot of **1**-symmetric cell.

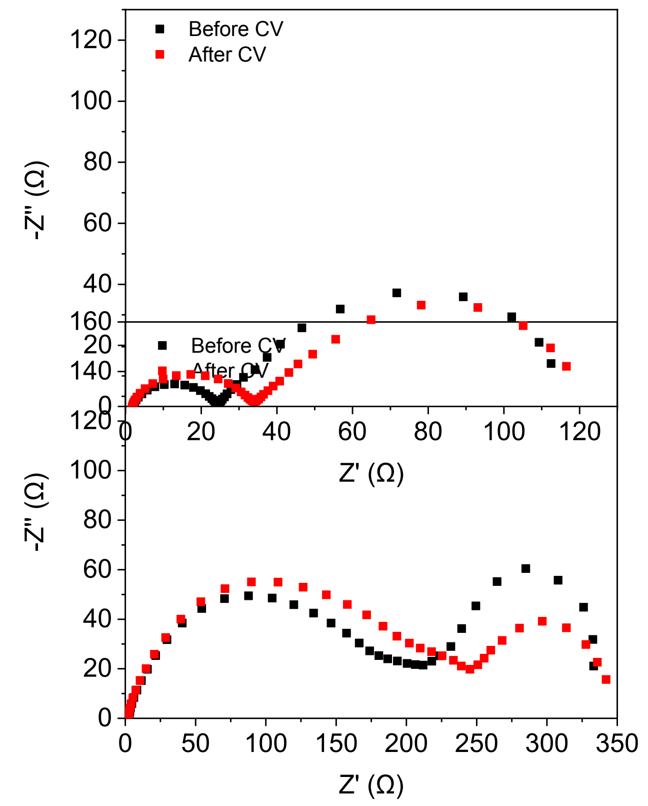

Figure S4. Nyquist plots of **3** (top) and **4** (bottom) symmetric cells before (black) and after (red) CV.

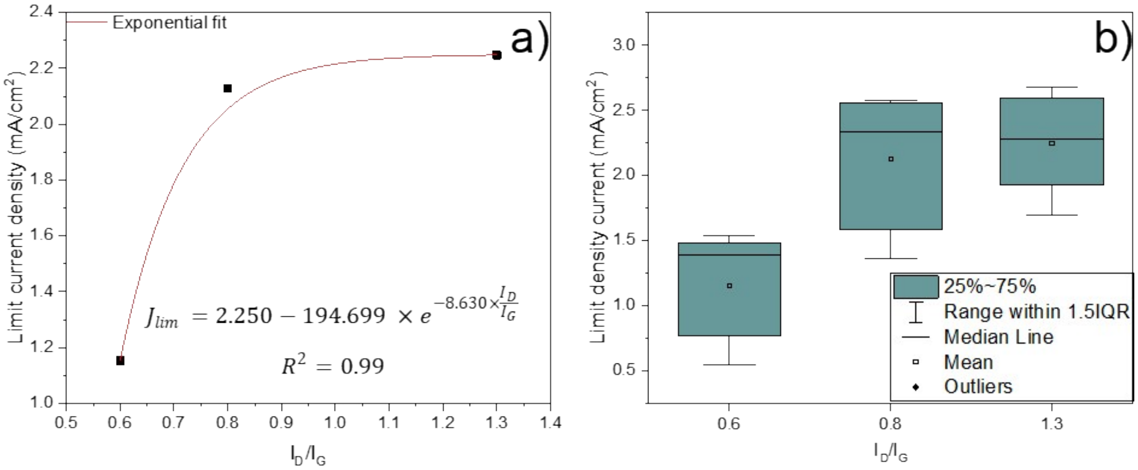

Figure S5. Exponential fit of the mean limit density current with I_D/I_G (a) and used statistics values (b).

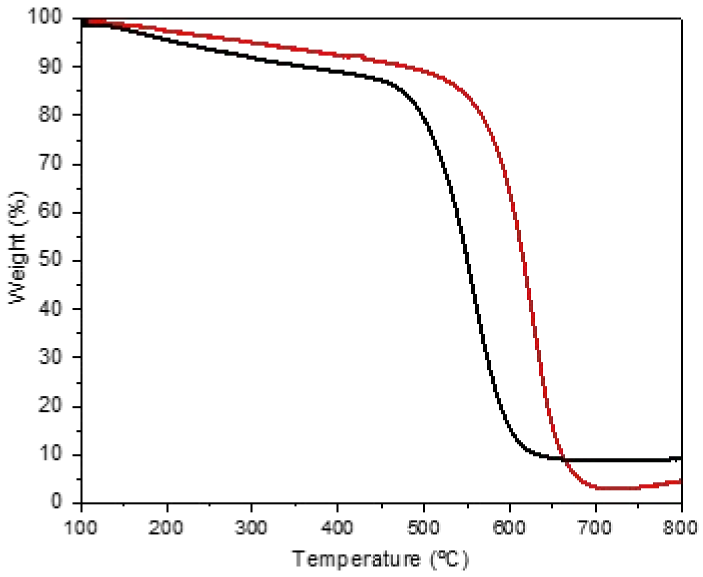

Figure S6. TGA of the CNTf-electrodes before (black; **1**) and after (red; **3-3**%) metal catalyst reduction.

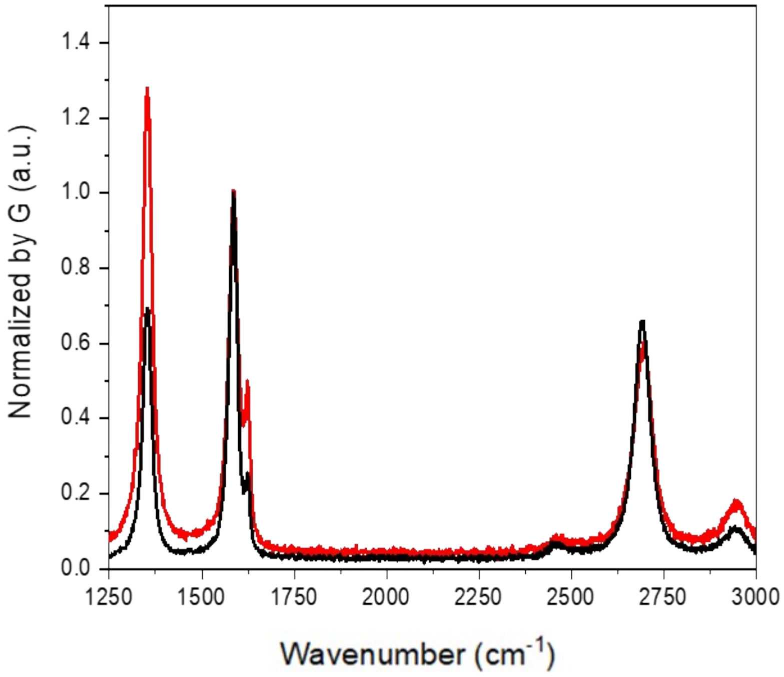

Figure S7. Raman spectra before (black; **1**) and after (red; **3-3%**) reduction of Fe catalyst.

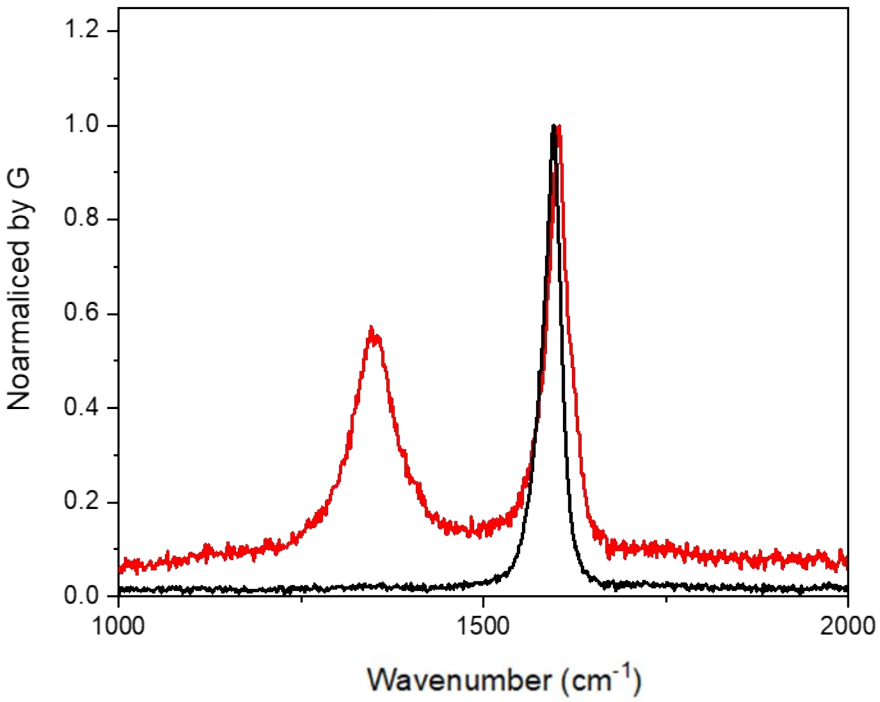

Figure S8. Raman spectra of commercial CNTf-electrodes without metal catalyst before (black) and after (red) ozonolysis functionalization.

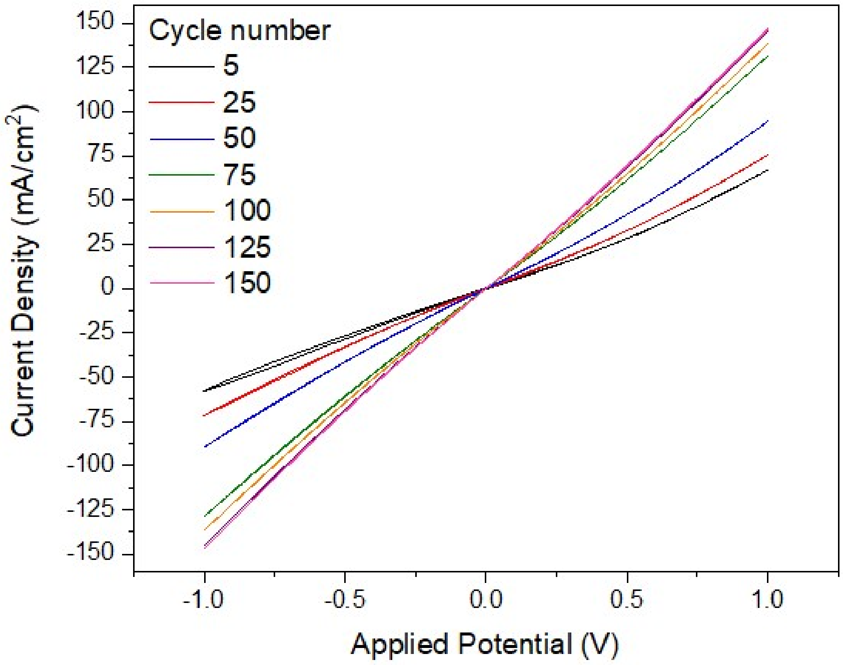

Figure S9. LSV of symmetric cells using a pristine commercial CNTf electrodes without catalyst.

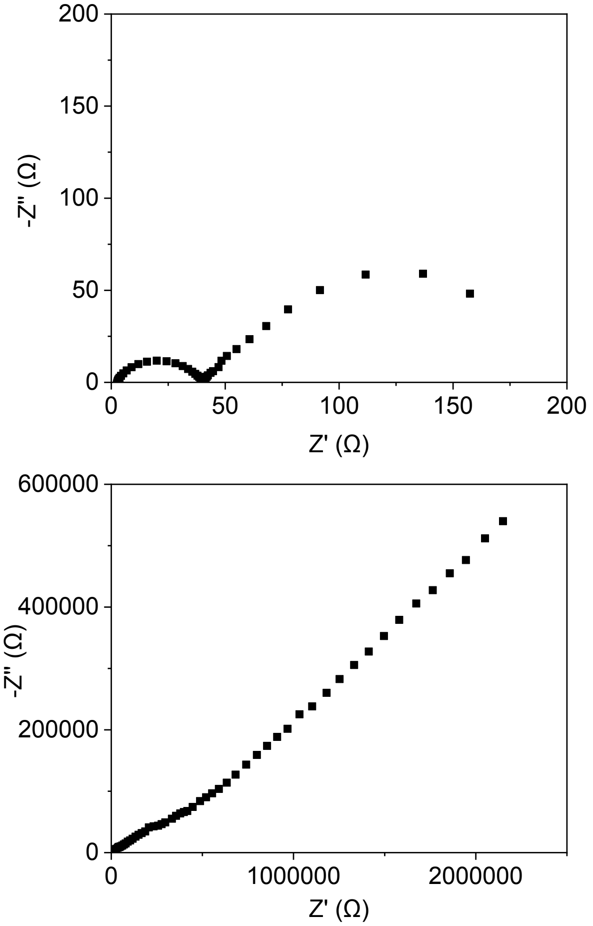

Figure S10. Nyquist plots of functionalized **3-3%** (top) and **3-0%** (bottom) symmetric cells.

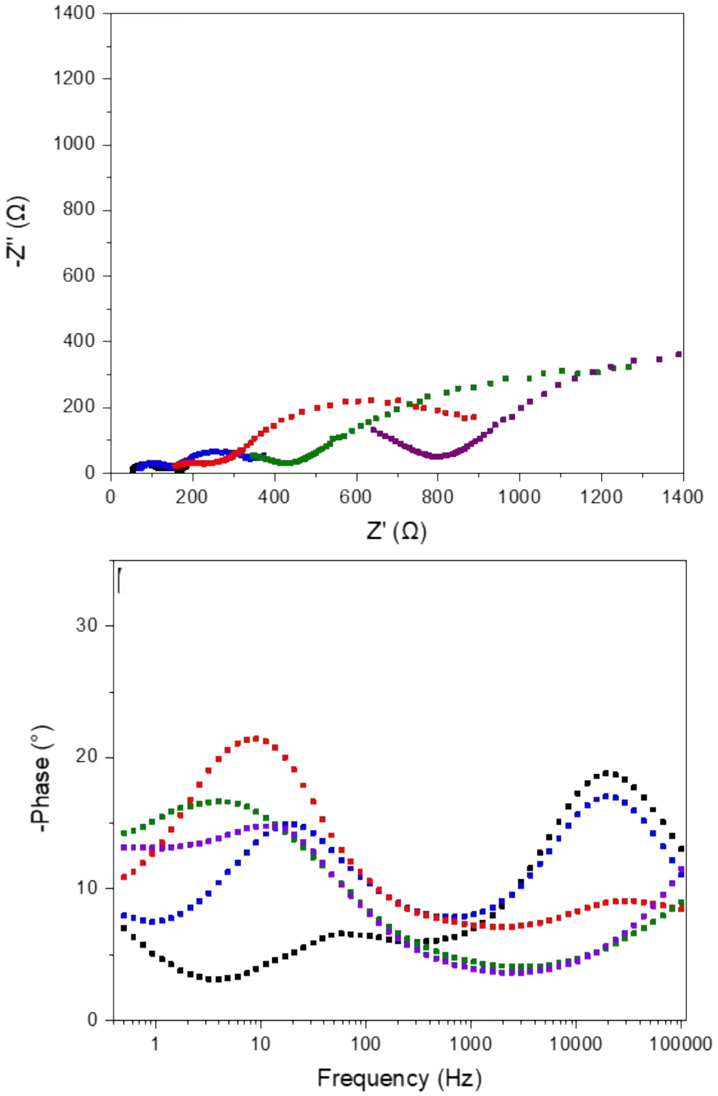

Figure S11. Nyquist (top) and Bode plots (bottom) of the DSSCs using **3**-CEs at different illumination conditions: 1 sun (black), 0.8 sun (blue), 0.6 sun (red), 0.4 sun (0.4), and 0.3 sun (purple).
